# The complete mitochondrial genome of wood-rotting fungus *Xylaria hypoxylon*

**DOI:** 10.1080/23802359.2019.1687025

**Published:** 2019-11-06

**Authors:** Huiying Zhou, Aifeire Abuduaini, Hong Xie, Ruiping Kang, Feiya Suo, Luodong Huang

**Affiliations:** a College of Life Science and Technology, Xinjiang University, Urumchi, China;; b Kunming Municipal Station of Forestry-Grassland Science and Technology Promotion, Kunming, Yunnan, China;; c College of Life Science and Technology, Guangxi University, Nanning, Guangxi, China

**Keywords:** *Xylaria hypoxylon*, wood-rotting fungus, mitochondrial genome, phylogenetic analysis

## Abstract

*Xylaria hypoxylo*n is a noticeable black fungus, and also habitual to cluster on rotting wood. In this study, the high-quality whole-genome of *X. hypoxylon* strain SFY20170806 was sequenced on the Illumina sequencing platform. The complete mitochondrial genome of *X. hypoxylon* was assembled and annotated. The single circular structure of 129,366 bp length is the largest species found in the order Xylariales. The overall GC content is 29.7% and gene composition includes 13 protein-coding genes (PCGs), 30 transfer RNA genes(tRNA), 2 ribosomal RNA genes(rRNA) and 6 open reading frames (ORF). Phylogenetic tree was constructed to validate the evolutionary relationship based on the complete mitogenomes from twelve taxa of four species of Xylariales, four species of Hypocreales, two species of Helotiales, one species of Microascales and *X. hypoxylon*. Phylogenetic analysis demonstrated that *X. hypoxylon* has a special evolutionary status and close genetic relationship with *Annulohypoxylon stygium*.

*Xylaria hypoxylon* belongs to the order Xylariales, which is one of the famous fungus widely distributed in temperate regions. It is usually buried in the soil on stumps or woody materials, and always distributed in a humid environment throughout Asia, Europe, and America (Stadler et al. 2014). A large number of researches have shown that *X. hypoxylon* fruit body and culture mycelial possess a variety of chemical compounds, such as inhibiting various pathogenic bacteria related to humans, inhibitory activity of HIV-1 reverse transcriptase, inhibiting the proliferation of various tumor cell lines and also as adjuvant to enhance the cellular immune response (Liu et al. 2006; Schüffler et al. 2007; Gu and Ding 2008; Hu et al. 2012; Kang et al. 2015; Canli et al. 2016). Therefore, *X. hypoxylon* is the pharmacological importance and considered to be a potential producer of medicine. Finally, based on this increasing interest, the complete mitochondrial genome of *X. hypoxylon* strain SFY20170806 was sequenced through Illumina sequencing technology.

The sample of *Xylaria hypoxylon* strain SFY20170806 was collected from Xishuangbanna in Yunnan Province, China (99°74′N, 22°.11′W). This voucher specimen (voucher no. SFY20170806) was deposited in the Herbarium of College of Life Science and Technology at Xinjiang University, Urumchi, China. The total genome DNA was extracted from the fruit body and culture mycelial of *X. hypoxylon* using MiniBEST Universal Genomic DNA Extraction Kit (Takara, Beijing, China). Then, the purified DNA was adopted to build 270 bp genomic library and sequenced with Illumina HiSeq2500 platform by Biomarker Technologies Corporation (Beijing, China). After the quality filtration of raw reads, the high-quality reads were assembled by SPAdes 3.9.0 (Bankevich et al. 2012). By comparing the sequence similarity with other Xylariales mitochondrial genomes, one contig of the mitochondrial DNA was identified. Finally, the mitochondrial genome annotation was performed by the UGENE ORFs finder, MFannot tool, AEWEN Web server, and combined with manual correction.

The complete mitochondrial genome sequence of the *X. hypoxylon* strain SFY20170806 was submitted to NCBI and assigned GenBank accession number MK574676. The length of the mitochondrial genome was 129,366 bp, containing 13 protein-coding genes (PCGs), 2 ribosomal RNA (rRNA) genes and 30 transfer RNA (tRNA) genes and 6 open reading frames (ORF298, ORF1059, ORF687, ORF581, ORF435, and ORF765). Furthermore, this mitochondrial genome contained large number of repeated sequences, introns, and intergenic regions. The overall base composition is 35.9% A, 34.4% T, 13.1% C, 16.6% G, with a CG content of 29.7%.

To validate the phylogenetic position of *X. hypoxylon* strain SFY20170806, its novel mitochondrial genome sequences, together with the 11 published and allied taxa sequences were downloaded from NCBI. Then, the 12 fungal species mitochondrial genomes were aligned by HomBlocks (Bi et al. 2018), resulting in alignments of each species with 9969 bp. The phylogenetic tree was conducted with MEGA7 (Kumar et al. 2016) and inferred by the maximum-likelihood method based on the General Time Reversible and Gamma distributed with invariant sites substitution model (GTR + G + I), with 1000 bootstrap replicates. Phylogenetic trees are displayed in Figure 1 and supported the fact that the Xylariales were more closely related to Helotiales than Microascales. Meanwhile, *X. hypoxylon* was clustered in the Xylariaceae clade and genetically close to *Annulohypoxylon stygium*.

**Figure 1.**
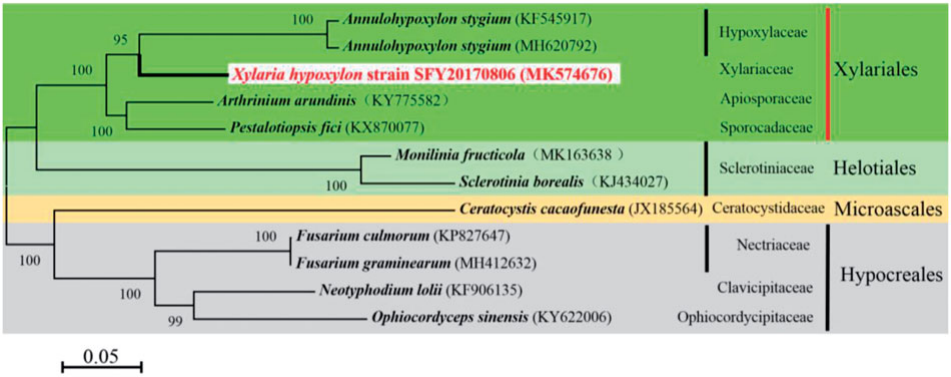
Phylogenetic analysis of mitochondrial genomes from *Xylaria hypoxylon* strain SFY20170806 and its related species. GenBank accession numbers are shown in parentheses.
